# A Novel Casual Video Game With Simple Mental Health and Well-Being Concepts (Match Emoji): Mixed Methods Feasibility Study

**DOI:** 10.2196/46697

**Published:** 2024-02-12

**Authors:** Russell Pine, James Mbinta, Lisa Te Morenga, Theresa Fleming

**Affiliations:** 1 School of Health, Victoria University of Wellington Wellington New Zealand; 2 Research Centre for Hauora and Health, Massey University Wellington New Zealand

**Keywords:** adolescent, anxiety, casual video games, digital mental health interventions, gaming, mental health, micro interventions, serious game, teenage, video game, youth

## Abstract

**Background:**

Adolescence is a crucial phase for early intervention and prevention of mental health problems. Casual video games are popular and have promise as a novel mechanism for reaching young people, but this potential has seldom been explored.

**Objective:**

This study aimed to explore the acceptability, feasibility, and possible indicators of therapeutic changes after playing a purpose-built novel casual video game (Match Emoji) with simple mental health and well-being content among young adolescents.

**Methods:**

We conducted a single-arm, nonrandomized trial of Match Emoji with 12- to 14-year-old school students (N=45; 26 [57%] New Zealand European, 12 [26%] Māori; 7 [15%] Asian or Pacific; 27 [60%] boys, 3 [6%] non-binary). Participants were invited to play Match Emoji for 15 minutes, 2-3 times a week over 2 weeks (a total of 60 minutes). Acceptability was assessed through the frequency and duration of use (analytics analyzed at the end of the 2-week intervention period and at weeks 4 and 6) and through participant reports. The Child and Adolescent Mindfulness Measure (CAMM), General Help-Seeking Questionnaire (GHSQ), Flourishing Scale (FS), and Revised Children’s Anxiety and Depression Scale (RCADS) were assessed at baseline and week 2 to indicate possible effects. Focus groups were held in week 4.

**Results:**

Most participants (n=39, 87%) used Match Emoji for at least 60 minutes over the 2-week intervention, with 80% (36/45) continuing to play the game after the intervention period. Mean change (from baseline to 2 weeks) on each measure was 1.38 (95% CI –0.03 to 2.79; *P*=.06) for CAMM; 0.8 (95% CI –2.71 to 4.31; *P*=.64) for GHSQ; –1.09 (95% CI –2.83 to 0.66; *P*=.21) for FS; and –3.42 (95% CI –6.84 to –0.001; *P*=0.49) for RCADS. Focus group feedback suggested that Match Emoji was enjoyable and helpful.

**Conclusions:**

The casual video game with mental health content appeared to be acceptable and provided a promising indication of possible therapeutic effects. This approach is worthy of further investigation.

**International Registered Report Identifier (IRRID):**

RR2-10.2196/31588

## Introduction

Mental distress and low well-being are common among adolescents [1-3] and appear to have increased over the past decade, at least in high-income nations [4-6]. Cognitive behavioral therapy (CBT) and psychotropic medications are recommended for young people experiencing mental health disorders [7,8]. Preventative programs that aim to buffer against higher levels of distress later in life also exist for young people [9]. Nevertheless, structural and attitudinal barriers inhibit access to mental health support for many young people [10].

Digital mental health interventions (DMHIs) refer to specialized content, support, or therapy for mental health conditions delivered electronically to treat, alleviate, or manage symptoms [11]. DMHIs encompass various technologies, including computerized CBT programs, chatbots, virtual reality for mental health conditions, games for mental health, apps, and interactive web pages [12]. Systematic reviews have shown promising effects for specific DMHIs across various age groups [13,14], such as CBT therapies for anxiety and depression [15,16]. Quality DMHIs can address some of the challenges often impacting face-to-face treatments [17,18]. For example, well-designed DMHIs can be used by young people irrespective of their level of distress, and they can be scaled up at a low cost due to their reduced reliance on clinically trained professionals [19,20].

Although this method of delivering mental health content is promising, engagement with DMHIs outside trial settings is typically lower than in trials [16]. Even playful interventions, such as Pesky gNATs [21] and SPARX [22], designed to appeal to young people’s interest in computer games, have had limited evidence of engagement [16]. In part, these findings may reflect mismatches between how end users engage with technology and the way tools are provided (eg, sessions approximating weekly face-to-face therapies may be a poor match with contemporary patterns of internet use) [16,23,24]. Moreover, a lack of appealing options, lack of trust, or uncertainty about digital tools for mental health purposes may create additional barriers [22-24]. Therefore, while DMHIs have a great capacity to address mental health needs, it is important to keep exploring new opportunities to improve engagement [19,25,26].

Casual video games (CVGs) refer to simple games that can be played in short bursts of time, require no specialized skills, are often used for relaxation [27] and distraction purposes [28], and are generally free or low-cost to download and play. Well-known CVGs include “Rise Up” and “Angry Birds.” “Rise Up” has been downloaded over 10 million times on the Google Play Store worldwide, and “Angry Birds” is played for approximately 200 million minutes daily [29,30]. Given their popularity and potential therapeutic effects, CVGs may be an approach that could be explored for delivering mental health and well-being content [28].

We systematically reviewed the effects of CVGs on anxiety, depression, stress, and low mood [31]. We found that 12 of the 13 trials reported promising results on their respective outcome measures. Following this work, we developed simple prototypes of CVGs with mental health concepts based on the puzzle, word, and match-3 genres and reviewed these in focus groups and interviews with young adolescents [32]. Young people indicated interest in this idea, with a match-3–style CVG being preferred. Subsequently, a game designer was contracted to develop the first version of Match Emoji*,* a simple match-3 CVG that includes brief text-based mental health and well-being messages, which have been previously described [33]. In brief, this includes short “micromessages,” which were developed using psychological well-being literature and were sometimes linked to gameplay, for example, “Great job focusing and matching the emojis!” and “Phew! Take a short breath to help focus again.” Subsequently, we held think-aloud interviews [34] with a small group of young adolescents to refine components.

In this study, we aimed to conduct a small open trial to explore the feasibility of using Match Emoji to strengthen the mental health and well-being of adolescents in a school setting. Findings from the study can help develop the literature on this new possible method for delivering mental health and inform processes for a possible future randomized controlled trial.

## Methods

### Design

The recruitment procedures, sample size, and analyses differed from those planned and published in our protocol paper [35] due to COVID-19 pandemic–related restrictions. Each departure from protocol is documented in the relevant section below.

This feasibility study used a mixed methods design. Adolescents attending New Zealand intermediate and high schools were recruited to participate in this study. They were shown how to use Match Emoji and then asked to play for 15 minutes, 2-3 times a week over 2 weeks (a total of 60 minutes). Analysis of game use, analytic data, and focus groups were held with all participants to explore the acceptability of Match Emoji. The therapeutic potential of the game was assessed by changes in mental health and well-being, which were assessed by 4 validated instruments.

### Recruitment

Before the onset of the COVID-19 pandemic, we developed a protocol to outline the guidelines for conducting the trial, including how participants would be recruited [35]. Initially, as 1 local secondary school had expressed interest in participating, we aimed to recruit students between the ages of 13 and 15 years from this school across 2-4 classrooms. However, several teachers had become ill during the recruitment phase, and the secondary school could no longer participate in the study. As such, we approached 2 secondary schools (students aged 12-18 years) and an intermediate school (students aged 12-14 years), which all expressed interest in participating in the study.

In the secondary school, we described the study to an assembly of over 400 students in years 9 and 10 (aged 12-14 years). Those interested in participating in the trial and with access to a smartphone or tablet were asked to take home information, an assent form, and a consent form for their parent or guardian. Of the 42 interested students, only 6 returned both forms. When recruiting participants in each intermediate school, the New Zealand government implemented restrictions on indoor face-to-face gatherings. At this time, indoor gatherings of up to 100 people were allowed. As such, instead of recruiting participants in an assembly, we delivered a 10- to 15-minute face-to-face presentation to students in each classroom, explaining the theory and research underpinning Match Emoji. In total, 39 returned the assent and parental consent forms. Given the primary aims, the inclusion criteria were students aged between 12 and 14 years who had access to a phone that could download Match Emoji and provided written consent from a parent or caregiver.

### Study Procedure

Consenting participants completed the Child and Adolescent Mindfulness Measure (CAMM), General Help-Seeking Questionnaire (GHSQ), Flourishing Scale (FS), and Revised Children’s Anxiety and Depression Scale (RCADS) at baseline. These assessments were completed in groups of 6 in the high school and 30 in the intermediate school. Students completed the questionnaires at their desks and were separated at least a meter apart from each other to protect privacy. Instructions on how to play and install the game were provided, and participants were given an opportunity to ask questions directly or email the primary researcher. Next, they were asked to play Match Emoji 2-3 times a week for a minimum of 15 minutes per session for 2 weeks (ie, a minimum of 60 minutes in total). Questions were repeated after the 2-week intervention period. All participants were invited to participate in focus groups held at each school 2 weeks later (4 weeks after the study began). After the study, *koha* (food and drink) was provided to acknowledge the student’s effort. No financial incentives or gifts were provided.

### The Intervention

**Figure 1:**
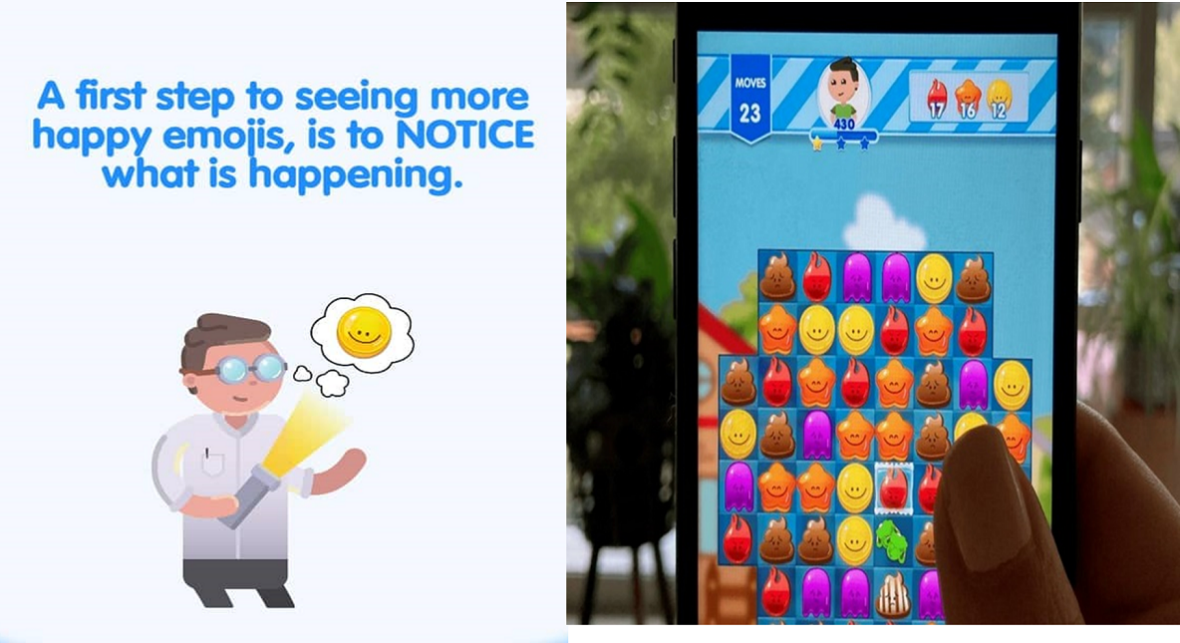
Screenshot of the Match Emoji video game.

The Match Emoji rationale, content, and processes have previously been described [33]. In brief, the micromessages in Match Emoji are based upon psychological well-being literature, specifically the *Five Ways to Wellbeing* [36]. As seen in Figure 1, these messages appear instead of in-game advertisements and function as prompts. For example, players are encouraged to read the message and practice skills including diaphragmatic breathing, noticing thoughts, or normalizing difficult emotions. In terms of the gameplay, users must identify and match 3 or more similar colored emojis together in rows or squares (a “match-3” game) to earn points. There are 6 different colored and shaped emojis, each representing an emotion or idea. The game has 99 levels, each designed to be completed within a few minutes, with a player advancing to the next level on completion of the current level. The gameplay becomes increasingly challenging as the player progresses.

### Measures and Outcomes

Demographic data were collected at baseline. Students who reported more than 1 ethnicity were categorized using the New Zealand Census ethnicity prioritization method [37].

Acceptability was assessed by the proportion of approached schools who agreed to participate, the number of participants who are able to download the game on their phone and those who fully participated in the study, and student feedback in focus groups. At week 4 (ie, after the intervention period), all participants were invited to take part in a 45-minute focus group at their school to explore their views of the intervention. Questions included (1) What parts of the game did you like? (2) What parts of the game could be improved? (3) What did you learn from playing the game? (4) Did you try and use any of the ideas from the game, and if so, which ones? and (5) Do you think you will continue to play Match Emoji? A general inductive approach was used to analyze the data from the focus groups [38]. The first author (RP) read participants’ responses several times to identify emerging themes and categories from the raw data. A research assistant read through the raw data to ensure the themes reflected the essence of the category. Appropriate quotes that conveyed the key core themes were recorded and integrated into the results. Lastly, game analytics for minutes played and the number of sessions were recorded on the Unity platform [39]. Unity is a secure platform for creating and operating interactive games.

The secondary outcome measures assessing therapeutic potential were changes from the pre- to postintervention (baseline and 2 weeks) time period on mental health and well-being domains calculated from the CAMM, a 10-item instrument measuring acceptance and mindfulness for use with children and adolescents aged 10 between 17 years; the GHSQ, which measures formal help-seeking intentions for nonsuicidal and suicidal problems; the 8-item FS, which measures self-perceived success in important areas such as relationships, self-esteem, purpose, and optimism as a single psychological well-being score; and the RCADS, a 47-item youth self-report questionnaire with subscales, such as separation anxiety disorder and generalized anxiety disorder.

The specific mental health and well-being domains assessed were mindfulness derived from the CAMM, help-seeking from the GHSQ, psychological well-being from the FS, and overall anxiety and depression score from the RCAD. Pretest and posttest summary statistics (mean, median, range, and SD) were computed using the R software (R Foundation for Statistical Computing) developer package. Data were assessed for normality using the Shapiro-Wilk normality test. Since data were not normally distributed, the nonparametric Wilcoxon signed rank test was used to compare the means between pairs of values (pre and post).

### Ethical Considerations

This study received ethics approval from the New Zealand Health and Disability Ethics Committee (21/NTA/34) on May 28, 2021. Data was de-identified and all participants provided informed consent. No financial compensation was provided to the study participants.

## Results

### Participants

Of the 45 adolescents who participated in the study (mean age 12.5, SD 0.33; range 12-14 years), 26 (57%) were New Zealand European, 12 (26%) were Māori, and 7 (15%) were Asian or Pacific. As seen in Table 1, the majority (n=27, 60%) were boys, while 15 (33%) were girls and 3 (6%) were nonbinary.

**Table 1:** Demographics of participants (N=45).

Characteristic		Value
**Age (years)**		
	Mean (SD)	12.5 (0.33)
	Range	12-14
**Gender, n (%)**		
	Boy	27 (60)
	Girl	15 (33)
	Nonbinary	3 (6)
**Ethnicity, n (%)**		
	Asian	3(6)
	Māori	12 (26)
	New Zealand European	26 (57)
	Pacific	4 (9)

### Acceptability

On average, each participant played 7.5 sessions for 24 minutes across the 2 weeks, comprising 180 minutes in total. In addition, data recorded from the focus groups suggested that, on average, participants completed 50 out of the 99 available levels during the 2-week duration of the study. Most participants in the focus groups said they would continue playing the game after completing the study. A total of 38 (84%) participants said they “would” continue to play the game, while 5 (11%) said they “might” continue to play. Only 2 (4%) participants said they would not continue to play the game after the trial. In addition, 36 (80%) reported playing Match Emoji after week 4, and 32 (71%) were still playing after week 6, according to game analytics from the Unity Platform. Findings from the focus groups suggested that participants enjoyed playing Match Emoji for several reasons. First, participants enjoyed the convenience of the game. For instance, many participants reported playing Match Emoji across multiple environments, including waiting rooms at the dentist, bus stops, and long car rides. As no internet connection was needed, participants could access the game whenever they wished. A participant explained, “I could play the game even when there was no Wi-Fi,” while another said, “The game was really good when waiting for appointments (be)cause it could distract me for a bit and didn’t use up data.”

Second, many participants reported enjoyment from playing the game. They described this enjoyment as stemming from game features such as increasing levels of challenge, the variety of emojis, and clear goals: “it was fun (be)cause the game got harder, but you knew what you had to do.” While there was some level of challenge, the simplicity of the game allowed students to bypass traditional barriers to CVGs, such as instructional videos. One participant described Match Emoji as a “super easy game to understand and play.” A smaller group of participants also provided suggestions about game features. This group appeared to be more frequent users of CVGs, as they provided recommendations based on other games they had played. One participant suggested, “You could add more rewards or more characters and then get more power-ups like Fortnite,” while another recommended, “coins, customization, themed music, and bonus rounds... add stuff like they have in other casual games.”

In general, participants liked the subtle aspect of accessing mental health content. As 1 participant mentioned, “the messages are a nice way of getting mental health information out there that isn’t in your face.” There was a high consensus that they preferred micromessages over typical in-game advertisements. However, some were initially skeptical about their value, “the messages were cringe at first but got way better.”

Of the intermediate and secondary schools approached to participate in the research, only 3 (25%) of the 12 took part in the study. Only 3 (7%) out of the 45 participants could not download Match Emoji onto their phones. In each case, this was because their phone had limited capacity to download the necessary software. All participants completed baseline and follow-up assessments, but several needed clarifications on wording related to the RCADS questionnaire items.

### Indicators of Possible Effects

As seen in Table 2, a small positive change was observed on the CAMM (mean difference 1.38, 95% CI –0.03 to 2.79) and on the RCADS (mean difference 3.42, 95% CI –6.84 to –0.001). In focus groups, when asked, “What did you learn from playing the game,” a number of participants answered that playing the game was helpful for their mental health and well-being: “I reckon playing the game for a bit of time was helpful for my mental health (be)cause it took my mind of stuff.” When asked, “Did you try and use any of the ideas from the game, and if so, which ones?” Several participants reported using specific skills suggested in Match Emoji: “Once when I started to think about annoying stuff, I tried the breathing thing, and it was actually pretty helpful,” and “I remember I got pretty mad at my brother and used the noticing a thought approach.”

**Table 2:** Changes in mental health and well-being indicators of adolescents aged between 12 and 14 years after 2 weeks of playing Match Emoji (N=45).

Outcome	Baseline, mean (SD)	Postintervention, mean (SD)	Mean differences (95% CI)	*P* value
CAMM^a^	22.44 (8.35)	23.82 (8.93)	1.38 (–0.03 to 2.79)	.06
GHSQ^b^	62.89 (21.96)	63.69 (23.30)	0.8 (–2.71 to 4.31)	.65
FS^c^	41.71 (11.58)	40.62 (12.07)	–1.09 (–2.83; 0.66)	.22
RCADS^d^	46.24 (26.39)	42.82 (26.49)	–3.42 (–6.84 to –0.001)	.049

^a^CAMM: Child and Adolescent Mindfulness Measure (mindfulness).

^b^GHSQ: General Help-Seeking Questionnaire (help-seeking).

^c^FS: Flourishing Scale (psychological well-being).

^d^RCADS: Revised Children’s anxiety and Depression Scale (overall anxiety and depression).

## Discussion

### Overview

In this study, we found that a CVG with psychological well-being concepts (Match Emoji) was a new and engaging mechanism of change that provided a promising indication of possible therapeutic impact. Most participants played more often and for a longer period than was requested for the study. Indeed, most participants continued to play in week 4. Small improvements in mindfulness assessed by CAMM and a small decrease in overall anxiety and depression assessed by RCADS were recorded. Given these promising changes, participants may have learned skills related to reducing their level of anxiety through playing Match Emoji. The findings of this small open feasibility trial indicate that the Match Emoji CVG was an acceptable way to support mental health and well-being in adolescents aged between 12 and 14 years.

Participants reported a high level of acceptability with Match Emoji, as evidenced by the game analytics, qualitative feedback, and the large portion of participants who were still playing the game in weeks 4 and 6. The percentage of participants who stated they continued to play Match Emoji even after week 6 of the study (n=32, 71%) is contrary to the poor retention rate typically found across the range of digital interventions. Real-world data on user engagement with popular mental health apps suggest that a small portion of users stay engaged with digital health interventions [16]. For example, once a health app is downloaded, approximately 4% of users continue to use the app after 15 days [24]. It is possible that the ongoing consultation with end users from the beginning of the development of Match Emoji, the simplicity with which CVGs can be played “on the go,” across environments with no Wi-Fi, and how playing CVGs fits with adolescents’ current behavior patterns may have been attributed to the high level of acceptability and engagement. That is, as many adolescents already play CVGs [32], there is less effort required to learn and change existing ways of engaging with technology. Data from the focus groups corroborated these findings. More specifically, participants mentioned they enjoyed playing for short periods across environments in comparison to computer games or those mobile phone games that require data to access. This is consistent with our previous work [32] and research [19], which suggests young adolescents tend to prefer brief therapeutic encounters. Moreover, Match Emoji enables large portions of the population to receive the same content irrespective of their proficiency with gaming or access to the internet, addressing a significant barrier to equity and engagement with DMHIs [11].

Our finding that most participants preferred micromessages over typical in-game advertisements is consistent with research assessing how in-game advertising in the form of short videos is distracting and can lead to disengagement, particularly among young people who often have a relatively short attention span [40,41]. Although paid versions can avoid advertisements, young people are reluctant to pay for them [41]. Thus, Match Emoji represents an opportunity for public health interventions to provide appealing free CVGs that replace the advertising with health-related micromessaging that is not distracting, annoying, or potentially harmful, as is the case with in-game advertising.

Similarly, diverse preferences were found with gamification elements of Match Emoji. The various preferences toward micromessages and game features among participants are consistent with the literature that suggests adolescents have different opinions about the type of DMHI they are attracted to [42,43]. Thus, while some adolescents may be frequent CVG users and interested in gamification elements, others may be less focused on these features and more attracted to learning about mental health and well-being [44]. In essence, opportunities to embed therapeutic processes within game elements are plentiful when researchers and game developers collaborate and are creative.

The protocol and implementation of this study were completed during the COVID-19–lined social distancing practices, which resulted in frequent changes to the restrictions on the size of inside gatherings and how educational facilities operated. Apart from the implications of the pandemic, 3 participants in the study could not download Match Emoji. This was because their phones lacked the storage capacity required to download the latest software and the game. Future research could use methods to compress digital mental health apps such as Match Emoji. In this way, the size of the app may better align with the capacity of users’ technology. In addition, some participants struggled to understand several questions on the RCADS. These questions were discussed in more detail with each participant to ensure they understood the meaning of each one. Despite these challenges, no significant issues occurred with conducting the study in a primary and intermediate school context.

### Limitations

There are limitations to this study; these include departures from the protocol due to COVID-19 impacts, which resulted in a small exploratory open trial only. There were also limited resources to conduct the study; this meant that the first author (RP) introduced the game to participants, led the recruitment process, supported the completion of assessments before and after playing the game, and facilitated the discussions about the game. Thus, the interpretation of the students’ feedback could be overly positive, and participants’ opinions and thoughts could be influenced by social pressures, including normative and informative conformity.

Further, the self-assessment outcome measures relied on the comprehension skills of young participants. While some participants raised their hands when unsure of a question, others may have merely guessed. Nevertheless, the 4 assessments appeared to be easy to implement in a short amount of time. Third, when recruiting participants at the secondary school, only 6 (14%) out of the 42 participants who signed the assent form returned their parental consent form for reasons unknown, suggesting that a different process is needed to recruit older adolescents for future trials. Lastly, students were not recruited based on their level of mental distress. Therefore, the results may have been affected by floor effects, whereby their mental health and well-being scores were already good or optimal and thus unlikely to improve any further. Despite these challenges and preliminary results, these findings are of interest as this is the first study to assess the feasibility of a co-designed CVG with psychological well-being concepts.

### Conclusion

Findings from this feasibility study suggest that Match Emoji, the purpose-built CVG with brief mental health messages, is promising as an acceptable and feasible approach for young adolescents. Future research should test clinical impacts through a randomized controlled trial. More broadly, the research also highlights the possibility of CVGs as a novel mechanism of delivering simple mental health and well-being messages.

## Abbreviations

CAMM: Child and Adolescent Mindfulness Measure
CBT: cognitive behavioral therapy
CVG: casual video game
DMHI: digital mental health intervention
FS: Flourishing Scale
GHSQ: General Health-Seeking Questionnaire
RCADS: Revised Children’s Anxiety and Depression Scale

## Data Availability

The data sets generated and analyzed during this study are available from the corresponding author on reasonable request.
